# Pediatric Electrocardiogram in Preparticipation Screening: Narrative Review of Normal Values in Key Features

**DOI:** 10.3390/children13020209

**Published:** 2026-01-31

**Authors:** Marianna Miliaraki, Ioannis Germanakis

**Affiliations:** 1 Pediatric Intensive Care, Medical School, University of Crete, 71300 Heraklion, Greece; 2 Pediatric Cardiology, Medical School, University of Crete, 71300 Heraklion, Greece

**Keywords:** electrocardiogram, pediatric, left ventricular hypertrophy, amplitudes, waveforms, normal values

## Abstract

**Highlights:**

- Published pediatric ECG normal values show substantial variability, particularly for parameters related to left ventricular hypertrophy, QTc prolongation and pre-excitation, despite consistent age-related trends.
- Greater standardization of ECG acquisition and measurement methods, alongside age-specific interpretation, is needed to improve reliability of pediatric ECG screening.

**Abstract:**

**Background**: Electrocardiography (ECG) represents an important noninvasive screening tool for heart disease in preparticipation screening of competitive athletes. However, interpretation of pediatric ECG based on age-specific reference values remains challenging, due to considerable variation among studies, influenced by population characteristics and documentation methodology. The variability of normal values in key pediatric ECG features regarding left ventricular hypertrophy (LVH), QTc prolongation and pre-excitation detection seem to have a significant impact on the efficacy of pediatric ECG as a preparticipation screening tool. **Aims and Scope of the Study**: This review aims to compare contemporary pediatric ECG reference ranges for key ECG features relevant to LVH, QTc, PR and QRS duration and highlight physiological and methodological sources of observed variability. **Methods**: A review of the current literature was conducted using common biomedical databases for studies reporting certain quantitative ECG reference values in healthy children from infancy through adolescence regarding the above selected key features. Reported values were summarized descriptively, with emphasis on developmental trends and methodological differences among studies affecting ECG values. **Results**: Across 16 pediatric studies, ECG parameters demonstrated consistent age-dependent developmental patterns, despite variability in absolute values. R-wave amplitudes in left precordial leads increased from infancy through early childhood and remained stable in older children, whereas S-wave amplitudes in right precordial leads showed greater variation between studies. PR intervals and QRS duration increased progressively with age across all datasets, while QTc values remained relatively stable throughout childhood and adolescence, with minimal sex-related differences. Variability in reported reference ranges was most pronounced for amplitude-based—compared to interval duration—parameters, and was influenced by differences in population characteristics, ECG acquisition techniques, and measurement methodology. **Conclusions**: This review summarizes contemporary ECG reference data in healthy children for the early detection of LVH, pre-excitation and QT prolongation, which are the main objectives of ECG screening in young athletes.

## 1. Introduction

Preparticipation cardiac screening refers to the early detection of potentially life-threatening conditions associated with sudden cardiac death in athletes and children, where coronary artery disease is virtually almost absent [1]. Early detection of cardiomyopathies (such as hypertrophic cardiomyopathy—HCM) and arrhythmogenic heart diseases, including Long QT (LQT) syndrome and pre-excitation syndromes such as Wolff–Parkinson–White (WPW), represent primary targets of preparticipation screening. ECG has become an integral screening tool in preparticipation screening in young asymptomatic competitive athletes in Europe, due to its established efficacy to detect left ventricular hypertrophy (LVH) through the use of appropriate amplitude criteria [2]. The detection of LQT and WPW is relatively straightforward, relying on automated ECG interval measurements by modern ECG interpretation software [3,4]. However, interpretation of pediatric ECG based on age-specific reference values remains challenging, due to considerable variation among studies, influenced by population characteristics and documentation methodology [5]. The variability of normal values in key pediatric ECG features regarding left ventricular hypertrophy (LVH), QTc prolongation and pre-excitation detection can have a significant impact on the efficacy of pediatric ECG as a preparticipation screening tool for the detection of HCM, LQT and WPW syndrome, respectively [4,6,7]. Traditionally, ECG amplitude criteria have been used to detect LVH in children. Thus, LVH is characterized by increased positive (R) amplitudes of QRS complex in left precordial leads (V5, V6) and increased negative (S) amplitudes of QRS complex in right precordial leads (V1, V2) [8,9]. Pre-excitation on ECG is characterized by a short PR interval, a delta wave, and QRS prolongation, reflecting ventricular activation through an accessory pathway [10]. Age-specific normal values for minimum PR and maximum QRS duration are of critical significance for the appropriate detection of pre-excitation, potentially associated with re-entry tachycardias or fast ventricular transmission of atrial tachycardias [11]. The documentation of prolonged QTc is a primary screening target of preparticipation screening, as long QT syndrome represents one of the most common inherited cardiac channelopathies, and is associated with a very high risk of sudden cardiac death in young athletes [12]. It can manifest as sudden cardiac death as early as infancy in affected families [13].

Interpretation of ECG in pediatric populations is complicated by rapid anatomical and developmental maturation. ECG parameters change substantially from infancy through adolescence, with changes in amplitude criteria related to LVH and age-specific changes in PR, QRS and QT duration, affecting the appropriate detection of LQT and WPW in pediatrics. As children grow, their cardiac size, thoracic geometry, body composition and autonomic tone evolve, influencing ECG voltages and morphology [14]. Normal developmental progression includes declining heart rate, increasing QRS duration, and alterations in S, R or T-wave amplitudes representing the transition from right- to left-ventricular predominance [15]. Classic reference datasets, including those by Davignon et al. [14] and Rijnbeek et al. [6], remain widely used but lack contemporary demographic diversity. More recent research work highlights ethnic ECG variabilities and supports the need for updated, population-representative reference values [5,7]. Another challenge in pediatric ECG interpretation concerns heart-rate correction of the QT interval (QTc), since the commonly used Bazett formula overcorrects the QT at high pediatric heart rates, leading to potential overdiagnosis of QT prolongation. Alternative formulas or demographic corrections have been reported, showing better performance [16].

Multiple methodological forms of heterogeneity among studies (manual or automated measurements, ECG filter and sampling rates, QT correction formulas) complicate between-study comparisons and the development of standardized pediatric normative ranges. Consequently, there is a need in refining age, gender-, and race-specific ECG values and improving QTc adjustment formulas [17]. Given these challenges, accurate, validated, pediatric ECG criteria are needed, as initial screening tools and as part of a multimodal framework, for early diagnosis of LVH, WPW and LQT syndrome, which are key targets of preparticipation screening in young athletes. The present narrative review seeks to consolidate available reference data for the main ECG parameters in healthy children, with particular emphasis on quantitative ECG indices relevant to these key targets of preparticipation screening.

## 2. Materials and Methods

### 2.1. Study Design

This narrative review synthesizes the existing literature reporting normal ECG values in healthy children from infancy through adolescence, emphasizing indices relevant to LVH, WPW and LQT detection. Developmental trends, methodological approaches, and reported reference values across contemporary pediatric studies were summarized and compared. Selection was guided by breadth of coverage and methodological approaches across studies.

### 2.2. Search Strategy and Sources

**Figure 1:**
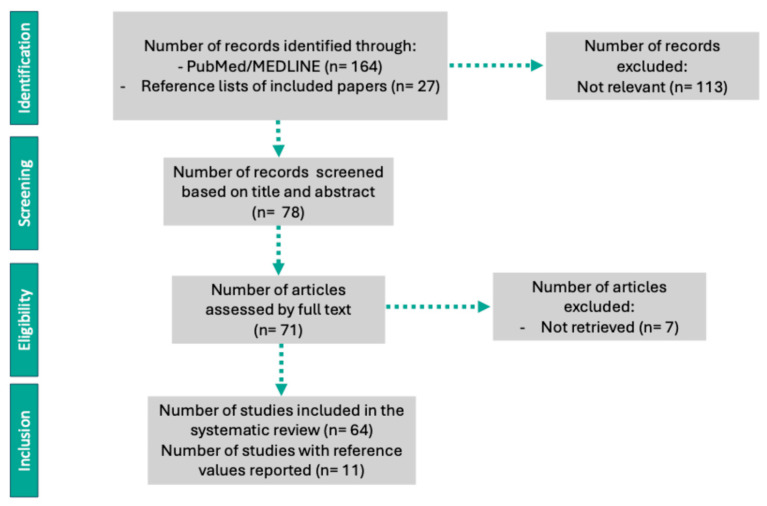
PRISMA-like flow diagram illustrating the study selection process.

A comprehensive search of the literature was performed primarily using PubMed/MEDLINE. Additional relevant studies were identified through manual screening of reference lists of included articles. The search was not restricted by publication year for inclusion. Search terms combined the following keywords: electrocardiography, normal values, pediatric, children, infants, adolescents, left ventricular hypertrophy, LVH criteria, and ECG amplitudes. The study selection process is illustrated in a PRISMA-like flow diagram (Figure 1).

### 2.3. Eligibility Criteria

Studies were included if they met the following criteria: (a) reported normal ECG measurements derived from healthy children (infants to ≤16 years); (b) provided quantitative data for LVH-, LQT, and/or WPW-related ECG indices (e.g., indices based on Sokolow–Lyon criteria, age-adjusted amplitude standards and interval durations); (c) used 12-lead ECGs recorded according to contemporary standard ECG methods; and (d) enrolled pediatric participants free from congenital heart disease, hypertension, or systemic conditions known to affect cardiac size or affect conduction (although commonly assessed these conditions through physical evaluation). Studies were excluded if their sample consisted of children with cardiac or systemic disease, lacked quantitative ECG values, or used unconventional lead placement and non-standard ECG recording techniques.

### 2.4. Data Extraction and Synthesis

For each eligible study, the following information was recorded: sample size, age distribution, ECG acquisition techniques, normative values for precordial lead amplitudes for R and S waves by age groups, QRS duration, QTc measurements and PR reference intervals. Attention was given to methodological differences affecting ECG voltages, such as equipment calibration, filter settings, and population ethnicity. Due to heterogeneity in study design, population, and reporting methodologies, formal meta-analysis was not performed. Instead, findings were synthesized descriptively, focusing on age-related developmental trends and pediatric ECG reference values, and methodological differences between studies. Values were summarized descriptively, both for studies reporting medians with interquartile ranges and those reporting means with standard deviations. Depending on the study, upper reference values were reported using either z-scores (98th or 99.5th percentile) or IQR-based cutoffs for the 98th percentile. For cross-study comparisons, descriptive averages were illustrated to provide an overview of central tendencies. These values are presented for comparative purposes only and should not be interpreted as pooled normative reference standards. All graphical displays and summary tables were generated using the statistical software SPSS (version 30), to visualize developmental trends and interstudy variability, rather than to report statistical significance.

## 3. Results

### 3.1. Included Studies

A total of 11 studies providing normative data were included. Table 1 summarizes the critical determinants of the resulting normative data, such as year of publication, demographic data of the reference populations, age distribution, methods used to exclude underlying heart disease, and ECG sampling rate.

### 3.2. LVH Criteria

**Figure 2:**
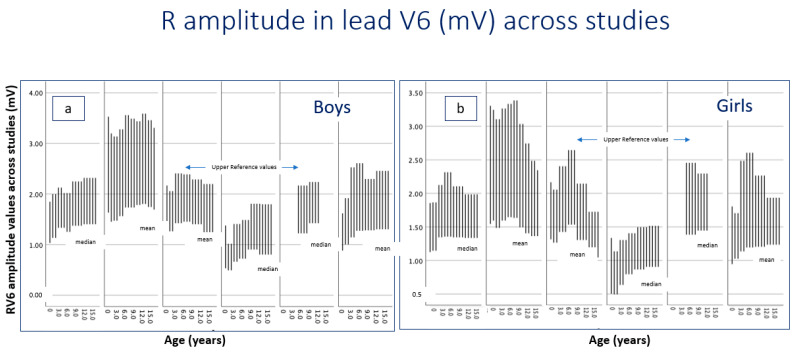
R-wave amplitude (mV) in lead V6 across reference studies [6,7,17,19,22,24]. Each plotted chart illustrates the reported distribution for each corresponding study. Lower values represent mean or median, while upper values represent the reported upper reference values. (**a**) Boys are displayed in the left panel, and (**b**) girls in the right panel. Methodological differences may contribute to interstudy variability.

As the detection of LVH as a primary target of preparticipation screening is based on detection of amplitude values exceeding the upper limit of normal, the following tables summarize the upper normal limits reported in all available studies. Across the reviewed studies, R-wave amplitudes in lead V6 demonstrated a clear age-related pattern, characterized by interstudy differences in absolute amplitude values, which were more pronounced than sex-related differences (Table 2 and Figure 2). In infancy (0.5–1 year), reported median or mean R-wave amplitudes ranged from 0.5 to 1.6 mV in both boys and girls, depending on the study and measurement approach. By early childhood, values increased modestly and typically clustered between 1.1 and 1.5. From approximately 3–7 years of age onward, most studies reported relatively stable amplitudes, commonly ranging between 1.3 and 1.8 mV in boys and 1.3 and 1.7 mV in girls. In older children (8–12 years), amplitudes remained in a similar range for most studies. In adolescence (13–16 years), no substantial further increase in R-wave amplitude was observed.

**Figure 3:**
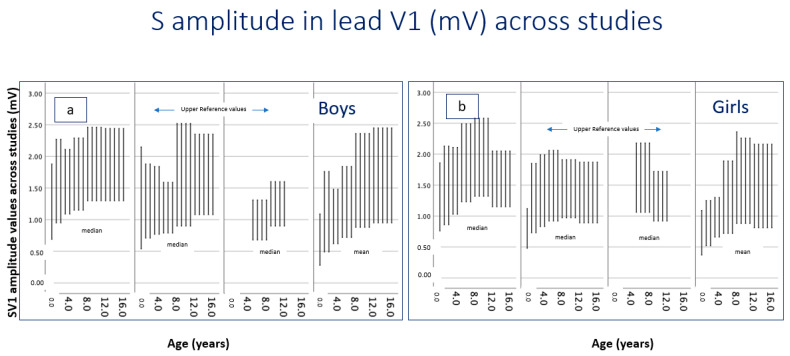
S-wave amplitude (mV) in lead V6 across reference studies [6,19,22,24]. Each plotted chart illustrates the reported distribution for each corresponding study. Lower values represent mean or median, while upper values represent the reported upper reference values. (**a**) Boys are displayed in the left panel, and (**b**) girls in the right panel. Methodological differences may contribute to interstudy variability.

S-wave amplitudes exhibited greater interstudy variability than R-wave amplitudes, reflecting differences in ECG acquisition techniques, correction formulas and population characteristics. Despite this dispersion in absolute values, a broadly consistent developmental pattern was observed across studies (Table 3 and Figure 3). S-wave amplitude increased during infancy and early childhood, reaching a plateau by mid-childhood, and remaining relatively stable through adolescence. Sex-related differences were minimal and inconsistently reported, with no uniform directional trend across age groups.

### 3.3. Pre-Excitation Detection in Pediatric ECG

Across all included studies, both minimum and maximum PR interval values demonstrated a gradual, age-dependent increase from infancy through adolescence. The overall developmental trajectory for minimum (Table 4) and maximum PR intervals (Table 5) was consistent across cohorts. Absolute PR interval ranges differed modestly between studies, but age-related trends were uniform. Sex-related differences in PR interval duration were minimal and generally within only a few milliseconds, with most studies reporting overlapping ranges for boys and girls across all age groups.

The reported upper limits of QRS duration increased progressively with age across all studies. Compared with other ECG intervals, QRS duration demonstrated greater interstudy variability, particularly between studies reporting means with standard deviations and those reporting medians with interquartile ranges. Sex-related differences were generally minimal in early childhood but became more apparent in adolescence in some cohorts, though absolute differences remained modest (Table 6). Variability in reported values was influenced by differences in ECG acquisition methods, signal filtering, and population characteristics.

### 3.4. LQT Detection in Pediatric ECG

QTc highest values demonstrated relatively narrow ranges across age groups and studies, compared with amplitude-based ECG parameters. Overall, QTc values remained relatively stable from infancy through adolescence, with no marked age-dependent increase (Table 7). Despite differences in methodology, the overall range of values was between 430 and 495 ms across all age groups and studies. Sex-related differences were minimal in early childhood, with some studies reporting slightly longer QTc values in girls during later childhood and adolescence, although these differences were not consistently observed across cohorts.

**Figure 4:**
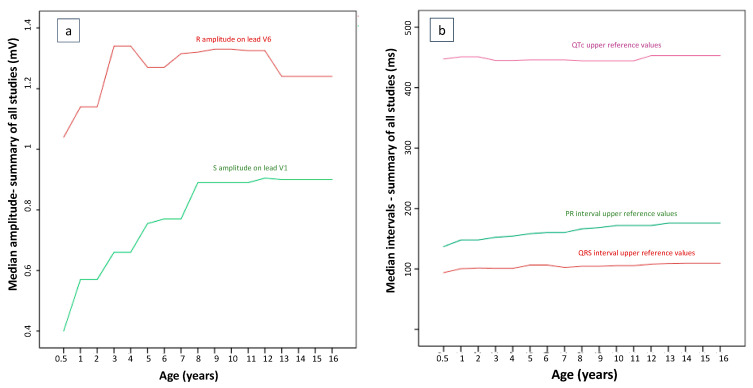
Developmental trends of (**a**) median pediatric ECG R-wave- (Lead V6) and S-wave-amplitude (Lead V1), and (**b**) median upper reference values of PR, QRS and QTc intervals calculated across studies. For visualization of developmental trends, age-specific ECG parameters were summarized using the median of reported central tendency measures across all included studies. This representation is for descriptive comparison only and does not represent pooled normative reference standards.

For a rough visualization of developmental trends, age-specific ECG parameters were summarized using the median of reported central tendency measures across all included studies (Figure 4).

## 4. Discussion

This narrative review summarizes contemporary studies reporting normative ECG values in healthy pediatric populations. It highlights the parameters most used and the complexity of assessing left ventricular hypertrophy (LVH) in children using electrocardiographic criteria. By comparing reference values across various studies and methodologies, this review illustrates both the developmental trends of ECG parameters and the substantial variability that complicates clinical interpretation. Historically, normative pediatric ECG values were derived from a few landmark studies, which have probable limitations (sampling rates and demographic differences) that might affect generalizability to current multi-ethnic pediatric populations. More recent studies are based on digital technologies and larger sample sizes to improve measurement consistency and update reference ranges [5].

A consistent finding across studies is the important influence of physiological maturation on pediatric ECG parameters. Infants typically show higher precordial amplitudes due to relatively right-dominant circulation, thinner chest walls, and different myocardial properties [25]. As children grow, ECG parameters change with growth, chest-wall configuration, autonomic tone, and the transition from right to left- ventricular dominance [15,26,27]. These developmental changes affect QRS waves, axis orientation and precordial lead amplitudes, making the creation of fixed amplitude cut-offs across all pediatric ages a difficult task [28]. Sex-related differences were generally small during prepubertal years and became clearer in adolescence, probably reflecting hormonal secretion and autonomic nervous system activity alterations. Moreover, this observation in puberty seems to concern repolarization parameters rather than QRS amplitudes [29,30].

Despite differences in study design, measurement techniques, and populations, all ECG parameters showed certain age-dependent trends across studies. However, despite similar developmental patterns, ECG values vary considerably across studies. Amplitude-based parameters (R- and S-wave amplitudes) showed greater variability and seem to be prone to methodological differences, whereas variability was less important for duration intervals, such as QRS and PR, which might prove more reliable in pediatric ECG interpretation, although PR and QTc interval differences have been reported as significant [5,6,31,32]. QTc values showed relatively narrow ranges across age groups, even though formulas like Bazett may lead to QT overcorrection at higher heart rates. Several studies support the concurrent use of alternative formulas, such as Fridericia, Framingham, or Hodges, which might exhibit less heart rate dependency [16,33,34,35]. However, very few studies have directly compared different QT correction formulas, and no single method has been validated for screening purposes in children. Consequently, pediatric ECG interpretation should take into account heart rate and the correction formula used, while borderline QTc findings should be interpreted with caution and confirmed with repeated ECGs.

Variability in ECG acquisition and methodological factors, including electrode placement, filtering, sampling rates, calibration, and manual versus automated measurement, likely affect measured voltages and contribute to differences in reported normal values [32,36]. Population characteristics, such as ethnicity, body habitus, and athletic status also influence ECG amplitudes, creating systematic differences between studies. Older reference standards were derived from homogeneous populations and analog ECG systems with broader technical variability, whereas more recent studies use digital ECGs and include various populations, providing more standardized outputs [5,37]. As a result, older normative values may not fully align with contemporary data [36], but they are still frequently used in clinical guidelines.

Based on the current literature, there are well-described age-related trends for amplitudes in precordial leads. This is reflected in ECG by relatively smaller R-waves and deeper S-waves in left precordial leads in early infancy compared to school age and adolescence [15]. The CHILDHEART study suggests that alternative indices (e.g., R in lead I and V6) or age thresholds perform better than traditional adult Sokolow–Lyon criteria [38]. The present review demonstrates that despite differences in reported R-wave amplitude in lead V6, the developmental trend is remarkably consistent across recent pediatric studies. All studies show increasing amplitudes during early childhood, followed by stabilization from mid-childhood through adolescence. The variability across studies may reflect population-level differences, variations in ECG equipment and calibration, electrode placement, or body habitus [39,40,41,42].

Developmental trends have also been reported with regard to QRS and QTc duration. In standard pediatric ECG interpretation studies, the upper limit of “normal” QRS duration is shorter in infants and increases with age [6,14,19]. A recent dataset of Japanese healthy children reported QRS means that increase with age, e.g., for 6-year-olds, mean QRS was ~85 ms (upper ~100 ms), and for 15-year-olds, mean QRS was ~99 ms [21]. A recent study of 1531 healthy children/adolescents (0–17 years) determined QTc distribution. The authors reported that a small percentage exceeded standard QTc thresholds (e.g., >440, 460, 480 ms), while the main sex differences were reported in adolescence, with girls having longer QTc than boys (mean ~454.1 ± 15.2 ms vs. ~438.3 ± 8.4 ms, respectively, *p* < 0.05) [43]. Another recent study reported that QTc varied by sex and race in older age groups [5].

A recent report indicated that ECG might be a statistically acceptable mass screening test for detecting LVH, LQT and WPW syndrome in children [44]. However, although ECG is widely accessible, its diagnostic performance for detecting LVH in children, even when interpreted with age-adapted criteria, seems to be limited due to age-dependent physiological changes, technical recording variables, and interindividual variability [21]. This complicates the use of universal cut-offs for LVH. A large institutional study covering 4637 children reported a high ECG sensitivity (>90%) but low specificity (43%) for LVH, whereas only a low proportion (17%) of LVH diagnoses were confirmed by echocardiography [9]. Another study in healthy children found weak correlations between ECG-based parameters for LVH and echocardiographic measurements of left ventricular mass and volume [45]. Classical LVH indices were originally derived from a few cohorts (e.g., the values of Davignon [14]) or adult populations (e.g., the Sokolow–Lyon criteria) and often overestimate LVH in children [46,47]. Amplitude-based criteria can be particularly misleading in thin or athletic children, who may have high precordial voltages in the absence of increased left ventricular mass [48]. On the other hand, children with obesity or certain ethnic populations may demonstrate attenuated voltages and mask true LVH [49]. These findings highlight the limitations of using ECG alone to diagnose LVH in children [45]. A combination of amplitude and non-amplitude ECG measures, adjusted for age and body size, might improve diagnostic performance [8]. The typical ECG findings for diagnosing WPW in children, such as a short PR interval, delta wave and wide QRS complex, also pose a similar challenge, due to developmental variability in cardiac anatomy, often necessitating additional electrophysiological confirmation [50]. Finally, a recent study demonstrated that, although a single resting ECG QTc might be important for initial detection, repeated ECGs or Holter monitoring appear to be better predictors of LQT syndrome [51]. Table 8 emphasizes situations in which borderline or abnormal ECG findings should prompt for repeat ECG, echocardiography, or referral to specialized pediatric cardiology settings, incorporating both amplitude and interval ECG criteria [6,51,52,53,54,55,56,57].

The present narrative review does not engage any meta-analytic methodologies. Differences in study design and ECG methodology limit direct quantitative comparison across studies. Therefore, the descriptive representations only show general trends and should not be interpreted as definitive normative reference values. Instead, they illustrate the developmental trends and the extent of variability between studies, leading to qualitative conclusions. These findings support the need for harmonized contemporary pediatric ECG reference ranges. More recent research emphasizes that ethnicity, body habitus, and athletic state influence amplitude parameters. This highlights the need for population-specific normal values and revision of international standards.

Overall, the present study highlights the heterogeneity of the available data and the differences among prior reports. Because the analysis is descriptive, this review does not propose new normative values but provides a structured foundation for future meta-analytic efforts and the development of standardized pediatric ECG charts. Normative ECG data should be age-, sex- and body-size-specific, and ideally derived from contemporary, well-characterized methodologies. The findings also highlight the need for updated, standardized, multicenter and multiethnic pediatric ECG reference datasets using uniform ECG acquisition protocols, and for LVH indices that reflect pediatric physiology. Priority should be given in the establishment of normal ECG values specifically related to the detection of hypertrophic cardiomyopathy [58], LQT syndrome [59] and WPW syndrome, when ECG is applied as a preparticipation screening tool in children participating in sports, or for large-scale screening of school-age children [60]. Modern athlete ECG frameworks recommend that both amplitude and non-amplitude criteria should be interpreted together. This combined approach is particularly important in children and adolescent athletes and may help reduce false-positive findings [61,62]. Future studies may engage machine learning tools, or computational models integrating amplitude parameters and waveform morphology, along with clinical and imaging variables to improve ECG diagnostic performance [63]. Inclusion of automated ECG analysis into electronic health records with decision support tools can greatly enhance the effort of screening for heart disease in pediatrics [64].

## 5. Conclusions

This review highlights the limited diagnostic performance of ECG amplitude criteria for detecting true LVH, WPW and LQT syndrome, main targets of preparticipation screening in pediatric populations. Given these limitations, a modern review of pediatric ECG normal values should (1) synthesize classic references, and describe their methodological limits; (2) present and compare newer percentile charts and large populations; (3) highlight the impact of QT correction methods and ECG acquisition parameters; (4) provide clinical recommendations; and (5) prioritize the creation of large, digital ECG studies and contemporary validation of normative values. The present review summarizes age-specific values related to LVH, WPW and LQT syndrome detection, based on the available literature, with the aim of assisting correct ECG interpretation when applied as a screening tool in young children participating in sports, and facilitating early detection of life-threatening conditions.

## Figures and Tables

**Table 1:** Methodological characteristics and sources of heterogeneity across included ECG studies.

	Publication Year	Race/ethnicity of children	Study sample	Ages (years)	Echo confirmation of LVH	Sampling rate (Hz)	Filter settings (Hz)	QT correction formulas	ECG acquisition method
**Citation** **⇣**									
[14]	1980	Caucasian (white)	2141	0–16	No	333	Not reported	Not reported	Auto & Visual *
[18]	1990	Caucasian (Scotland, UK)	1780	0–16	No	500	Not reported	Not reported	Automated
[6]	2001	Caucasian (predominantly)	1912	0–16	No	1200	~320	Not reported	Auto & Visual *
[19]	2008	Caucasian (Turkey)	2241	0–16	No	500	0.05–150	Bazett	Automated
[20]	2018	Caucasian (Netherlands)	1011	0–18	No	250–500	Automatic (not specified)	Bazett	Automated
[21]	2018	Asian (Japanese)	16,773	6–15	No	500	0.5–35	Custom vs Bazett	Auto & Visual *
[22]	2019	Caucasian (Turkey)	1163	0-16	No	500	Not reported	Bazett	Auto & Visual *
[7]	2020	North American, mixed race	23,064	0–18	No	500	Unfiltered	Bazett, Framingham, Friderica	Automated
[23]	2021	Eurasian, mixed race	12,512	5–18	Yes	4000	≤150	Bazett	Automated
[24]	2022	Caucasian (Polish)	336	5–16	No	1000	Not reported	Bazett	Auto & Visual *
[17]	2023	Asian (Taiwanese)	1884	0–18	No	500	Not reported	Not reported	Auto & Visual *

Key methodological, demographic and screening characteristics of all included studies providing ECG normative data from pediatric series [6,7,14,17,18,19,20,21,22,23,24]. * Visual verification of automated ECG measurements by a cardiologist.

**Table 2:** Cross-study comparison of pediatric upper reference values of R-wave amplitude in V6.

**Citation** **⇢**	[14]	[6]		[7]		[17]		[22]		[24]		[19]		[21]	
	98th percentile	98th percentile		99.5th percentile		98th percentile		98th percentile		98th percentile		98th percentile		98th percentile	
**Age (years)** **⇣**		Boys	Girls	Boys	Girls	Boys	Girls	Boys	Girls	Boys	Girls	Boys	Girls	Boys	Girls
0.5–1	2.0	1.84	1.85	3.52	3.3	2.16	2.16	1.37	1.33			1.61	1.8		
1	2.4	1.99	1.86	3.19	3.24	2.05	2.05	1.01	1.13			1.91	1.7		
2	2.4	1.99	1.86	3.13	3.10	2.05	2.05	1.01	1.13			1.91	1.7		
3	2.4	2.12	2.12	3.13	3.10	2.4	2.4	1.4	1.3			2.52	2.48		
4	2.4	2.12	2.12	3.27	3.26	2.4	2.4	1.4	1.3			2.52	2.48		
5	2.4	2.01	2.31	3.27	3.26	2.4	2.4	1.4	1.3	2.16	2.45	2.6	2.6		
6	2.4	2.01	2.31	3.55	3.33	2.38	2.64	1.48	1.4	2.16	2.45	2.6	2.6	2.29	2.25
7	2.4	2.01	2.31	3.55	3.33	2.38	2.64	1.48	1.4	2.16	2.45	2.6	2.6		
8	2.4	2.24	2.10	3.48	3.38	2.38	2.64	1.48	1.4	2.16	2.45	2.29	2.26		
9	2.4	2.24	2.10	3.48	3.38	2.28	2.14	1.80	1.49	2.23	2.29	2.29	2.26		
10	2.4	2.24	2.10	3.43	3.03	2.28	2.14	1.80	1.49	2.23	2.29	2.29	2.26		
11	2.4	2.24	2.10	3.43	3.03	2.28	2.14	1.80	1.49	2.23	2.29	2.29	2.26		
12	2.2	2.31	1.98	3.58	2.74	2.28	2.14	1.80	1.49	2.23	2.29	2.45	1.93	2.52	2.02
13	2.2	2.31	1.98	3.58	2.74	2.19	1.72	1.79	1.51			2.45	1.93		
14	2.2	2.31	1.98	3.45	2.48	2.19	1.72	1.79	1.51			2.45	1.93		
15	2.2	2.31	1.98	3.45	2.48	2.19	1.72	1.79	1.51			2.45	1.93	2.55	1.9
16	2.2	2.31	1.98	3.3	2.34	2.19	1.72	1.79	1.51			2.45	1.93		

Reported upper reference values are presented as mean +2 SD (parametric data) or IQR-based cut-off (non-parametric data), which correspond to different distribution percentiles (98th or 99.5th) depending on the original study [6,7,14,17,19,21,22,24]. Values are shown for descriptive comparison only and should not be interpreted as pooled normative reference standards.

**Table 3:** Cross-study comparison of pediatric upper reference values of S-wave amplitude in V1.

**Citation** **⇢**	[14]	[6]		[19]		[22]		[24]		[21]		[17]	
	98th percentile	98th percentile		98th percentile		98th percentile		98th percentile		98th percentile		98th percentile	
**Age (years)** **⇣**		Boys	Girls	Boys	Girls	Boys	Girls	Boys	Girls	Boys	Girls	Boys	Girls
0.5–1	0.9	1.88	1.86	1.09	1.09	2.15	1.12					0.94	0.94
1	0.8	2.27	2.13	1.76	1.25	1.88	1.85					1.25	1.25
2	0.8	2.27	2.13	1.76	1.25	1.88	1.85					1.25	1.25
3	0.8	2.11	2.11	1.48	1.3	1.84	1.99					1.25	1.25
4	0.8	2.11	2.11	1.48	1.3	1.84	1.99					1.25	1.25
5	0.8	2.29	2.49	1.84	1.89	1.59	2.06	1.31	2.18			1.25	1.25
6	0.8	2.29	2.49	1.84	1.89	1.59	2.06	1.31	2.18	2.25	2.29	1.56	1.38
7	0.8	2.29	2.49	1.84	1.89	1.59	2.06	1.31	2.18			1.56	1.38
8	0.8	2.46	2.58	2.36	2.36	2.52	1.91	1.31	2.18			1.56	1.38
9	0.8	2.46	2.58	2.36	2.26	2.52	1.91	1.6	1.72			1.49	1.43
10	0.8	2.46	2.58	2.36	2.26	2.52	1.91	1.6	1.72			1.49	1.43
11	0.8	2.46	2.58	2.36	2.26	2.52	1.91	1.6	1.72			1.49	1.43
12	0.8	2.44	2.05	2.45	2.16	2.35	1.87	1.6	1.72	2.57	2.22	1.49	1.43
13	0.8	2.44	2.05	2.45	2.16	2.35	1.87					1.5	1.5
14	0.8	2.44	2.05	2.45	2.16	2.35	1.87					1.5	1.5
15	0.8	2.44	2.05	2.45	2.16	2.35	1.87			2.87	1.97	1.5	1.5
16	0.8	2.44	2.05	2.45	2.16	2.35	1.87					1.5	1.5

Reported upper reference values correspond to different percentile definitions (98th or 99.5th) depending on the original study [6,14,17,19,21,22,24]. Values are shown for descriptive comparison only and should not be interpreted as pooled normative reference standards.

**Table 4:** Comparative lower reference values of the PR intervals in children and adolescents.

**Citation** **⇢**	[6]		[7]		[23]		[19]		[22]		[17]		[21]	
	2nd percentile		0.5th percentile		2nd percentile		2nd percentile		2nd percentile		2nd percentile		2nd percentile	
**Age (years)** **⇣**	Boys	Girls	Boys	Girls	Boys	Girls	Boys	Girls	Boys	Girls	Boys	Girls	Boys	Girls
0.5–1	82	88	79	77			78	70	72	72	91	91		
1	86	78	80	79			75	76	65	72	104	104		
2	86	78	84	83			75	76	65	72	104	104		
3	98	99	84	83			86	84	84	82	107	107		
4	98	99	90	87			86	84	84	82	107	107		
5	99	92	90	87	100	98	97	91	89	89	107	107		
6	99	92	92	89	100	98	97	91	89	89	101	99	96	96
7	99	92	92	89	100	98	97	91	89	89	101	99		
8	105	103	91	94	102	100	90	90	92	94	101	99		
9	105	103	91	94	102	100	90	90	92	94	105	102		
10	105	103	95	94	102	100	90	90	92	94	105	102		
11	105	103	95	94	102	100	90	90	92	94	105	102		
12	107	106	87	91	106	104	92	100	100	98	105	102	103	103
13	107	106	87	91	106	104	92	100	100	98	101	106		
14	107	106	90	92	106	104	92	100	100	98	101	106		
15	107	106	90	92	106	104	92	100	100	98	101	106	107	106
16	107	106	91	93	112	112	92	100	100	98	101	106		

Reported lower reference values correspond to different percentile definitions (0.5th or 2nd) depending on the original study [6,7,17,19,21,22,23]. Values are presented for descriptive comparison only and do not represent pooled normative reference standards.

**Table 5:** Comparative upper reference values of the PR intervals in children and adolescents.

**Citation** **⇢**	[6]		[7]		[23]		[19]		[22]		[17]		[21]	
	98th percentile		99.5th percentile		98th percentile		98th percentile		98th percentile		98th percentile		98th percentile	
**Age (years)** **⇣**	Boys	Girls	Boys	Girls	Boys	Girls	Boys	Girls	Boys	Girls	Boys	Girls	Boys	Girls
0.5–1	141	133	150	148			122	128	118	128	145	145		
1	151	147	151	150			146	158	147	146	148	148		
2	151	147	156	155			146	158	147	146	148	148		
3	152	153	156	155			150	158	156	140	151	151		
4	152	153	157	160			168	172	156	140	151	151		
5	160	156	157	160	160	158	168	172	151	161	151	151		
6	160	156	161	160	160	158	168	172	151	161	163	160	159	158
7	160	156	161	160	160	158	172	178	151	161	163	160		
8	174	163	170	162	168	164	172	178	204	151	163	160		
9	174	163	170	162	168	164	172	178	204	151	163	167		
10	174	163	172	173	168	164	172	178	204	151	163	167		
11	174	163	172	173	168	164	172	178	170	184	163	167		
12	178	176	155	154	174	172	184	174	170	184	163	167	173	177
13	178	176	155	154	174	172	184	174	170	184	179	176		
14	178	176	157	157	174	172	184	174	170	184	179	176		
15	178	176	157	157	174	172	184	174	170	184	179	176	186	186
16	178	176	163	160	184	176	184	174	170	184	179	176		

Reported upper reference values correspond to different percentile definitions (98th or 99.5th) depending on the original study [6,7,17,19,21,22,23]. Values are presented for descriptive comparison only and do not represent pooled normative reference standards.

**Table 6:** Upper reference values of QRS duration in boys and girls: summary of studies.

**Citation** **⇢**	[6]		[7]		[23]		[20]		[19]		[22]		[17]		[21]	
	98th percentile		99.5th percentile		98th percentile		98th percentile		98th percentile		98th percentile		98th percentile		98th percentile	
**Age (years)** **⇣**	Boys	Girls	Boys	Girls	Boys	Girls	Boys	Girls	Boys	Girls	Boys	Girls	Boys	Girls	Boys	Girls
0.5–1	86	80	101	107			303	303	94	90	92	94	98	98		
1	88	85	105	101			303	303	100	100	92	106	106	106		
2	88	85	115	103			322	322	100	100	92	106	106	106		
3	92	88	115	103			322	322	98	98	100	102	106	106		
4	92	88	125	114			348	348	98	98	100	102	108	108		
5	98	95	125	114	104	98	348	348	108	100	99	105	108	108		
6	98	95	118	115	104	98	371	371	108	100	99	105	108	108	100	95
7	98	95	118	115	104	98	371	371	108	100	99	105	118	100		
8	103	99	122	115	106	102	371	371	106	100	100	116	118	100		
9	103	99	122	115	106	102	371	371	106	100	100	116	118	100		
10	103	99	119	116	106	102	395	395	106	100	100	116	107	105		
11	103	99	119	116	106	102	395	395	106	100	100	116	107	105		
12	111	106	123	110	108	110	395	395	108	108	102	114	107	105	109	103
13	111	106	123	110	108	110	395	395	108	108	102	114	120	108		
14	111	106	124	112	108	110	414	414	108	108	102	114	120	108		
15	111	106	124	112	108	110	414	414	108	108	102	114	120	108	115	105
16	111	106	124	114	114	106	426	426	108	108	102	114	120	108		

Reported upper reference values correspond to different percentile definitions (98th or 99.5th) depending on the original study [6,7,17,19,20,21,22,23]. These values are for descriptive comparison only and should not be interpreted as pooled normative standards.

**Table 7:** Pediatric QTc upper reference values by age and sex from multiple studies.

**Citation** **⇢**	[6]		[7]		[23]		[20]		[19]		[22]		[17]	
	98th percentile		99.5th percentile		98th percentile		98th percentile		98th percentile		98th percentile		98th percentile	
**Age (years)** **⇣**	Boys	Girls	Boys	Girls	Boys	Girls	Boys	Girls	Boys	Girls	Boys	Girls	Boys	Girls
0.5–1	449	446	499	499			432	432	489	482	429	435	334	334
1	455	447	491	489			432	432	490	480	441	436	363	363
2	455	447	492	487			426	426	490	480	441	436	363	363
3	448	442	492	487			426	426	483	470	431	427	374	374
4	448	442	494	484			427	427	483	470	431	427	374	374
5	443	449	494	484	465	463	427	427	464	465	433	435	374	374
6	443	449	487	484	465	463	431	431	464	465	433	435	386	384
7	443	449	487	484	465	463	431	431	464	465	433	435	386	384
8	440	447	488	483	465	467	431	431	474	486	441	442	386	384
9	440	447	488	483	465	467	431	431	474	486	441	442	383	379
10	440	447	485	492	465	467	435	435	474	486	441	442	383	379
11	440	447	485	492	465	467	435	435	474	486	441	442	383	379
12	449	457	486	488	464	468	435	435	473	486	433	436	383	379
13	449	457	486	488	464	468	435	435	473	486	433	436	403	412
14	449	457	481	487	464	468	440	440	473	486	433	436	403	412
15	449	457	481	487	464	468	440	440	473	486	433	436	403	412
16	449	457	480	490	454	466	440	440	473	486	433	436	403	412

Reported upper reference values correspond to different percentile definitions (98th or 99.5th) depending on the original study [6,7,17,19,20,22,23]. QTc values were calculated using different correction formulas, which may influence reported ranges.

**Table 8:** Practical pediatric ECG screening, follow-up and referral recommendations.

ECG Finding	Borderline Values	Recommended Follow-Up	References
Left Ventricular Hypertrophy (LVH)	Precordial R- or S-wave amplitude near the 98th percentile (age/sex-specific values)	- Repeat ECG to confirm persistence - Consider echocardiography - Referral to specialized pediatric cardiology settings for persistent LVH	[6,52]
QTc Prolongation (Long QT Syndrome)	Borderline: 440–460 ms Prolonged: >460 ms High suspicion: >480 ms (age/sex-specific values)	- Repeat ECG to confirm persistence - Consider Holter monitoring - Referral to specialized pediatric cardiology settings for QTc >460ms in children, especially if associated with symptoms or family history	[51,54,55]
Pre-excitation/ WPW Pattern	Short PR interval with delta wave, and normal or prolonged QRS (age/sex-specific values)	- Confirm pattern on repeat ECG - Consider Holter monitoring to assess loss of pre-excitation or conduction properties - Referral to pediatric electrophysiology settings if pattern persists, especially if symptomatic or if family history of sudden cardiac death	[53,56,57]

## Data Availability

No new data were created.

## Abbreviations

The following abbreviations are used in this manuscript:

ECG	Electrocardiography
LVH	Left Ventricular Hypertrophy
LQT	Long QT syndrome
WPW	Wolff–Parkinson–White Syndrome
mV	millivolts
ms	milliseconds
